# Clinical significance of regional constructive and wasted work in patients receiving cardiac resynchronization therapy

**DOI:** 10.3389/fcvm.2024.1301140

**Published:** 2024-03-06

**Authors:** Chun-Li Wang, Lung-Sheng Wu, Chia-Tung Wu, Yung-Hsin Yeh, Yu-Wen Cheng, Kun-Chi Yen, Yi-Hsin Chan, Chi Chuang, Chi-Tai Kuo, Pao-Hsien Chu

**Affiliations:** ^1^ Division of Cardiology, Department of Internal Medicine, Chang Gung Memorial Hospital, Linkou, Taiwan; ^2^ College of Medicine, Chang Gung University, Taoyuan, Taiwan; ^3^ Microscopy Core Laboratory, Chang Gung Memorial Hospital, Linkou, Taiwan; ^4^ Division of Cardiology, Department of Internal Medicine, New Taipei City Municipal Tucheng Hospital, New Taipei City, Taiwan

**Keywords:** cardiac resynchronization therapy, myocardial work, constructive work, wasted work, reverse remodeling, survival, heart failure

## Abstract

**Background:**

Previous studies have shown that global constructive work (CW) and wasted work (WW) predict response to cardiac resynchronization therapy (CRT). This study evaluated the predictive value of regional CW and WW for reverse remodeling and clinical outcomes after CRT.

**Methods:**

We performed a prospective study involving 134 CRT candidates with left bundle branch block and left ventricular ejection fraction ≤35%. Global and regional CW and WW were calculated using pressure-strain loop analysis. CRT response was defined by reverse remodeling as a reduction of ≥15% in left ventricular end-systolic volume after six months.

**Results:**

At six-month follow-up, 92 (69%) patients responded to CRT. Of the regional CW and WW measures, lateral wall (LW) CW and septal WW were most strongly and significantly correlated with reverse remodeling. At multivariate analysis, LW CW and septal WW were both independent determinants of reverse remodeling. When LW CW and septal WW were included in the model, global CW and WW were not independently associated with reverse remodeling. LW CW and septal WW predicted reverse remodeling with an area under the curve (AUC) of 0.783 (95% CI: 0.700–0.866) and 0.737 (95% CI: 0.644–0.831), respectively. Using both variables increased the AUC to 0.832 (95% CI: 0.755–0.908). Both LW CW ≤878 mmHg% (HR 2.01; 95% CI: 1.07–3.79) and septal WW ≤181 mmHg% (HR 2.60; 95% CI: 1.38–4.90) were significant predictors of combined death and HF hospitalization at two-year follow-up.

**Conclusion:**

LW CW and septal WW before CRT are important determinants of reverse remodeling and clinical outcomes.

## Introduction

Cardiac resynchronization therapy (CRT) reduces morbidity and mortality in patients with heart failure (HF) and a wide QRS complex (1). However, a significant portion of patients who receive CRT do not respond favorably to the therapy (2). Several echocardiographic measures have been suggested to predict CRT response by analyzing the timing of mechanical events (3–5). Although these time-delay parameters initially showed promise, randomized controlled trials have shown that these parameters are not reliable predictors of CRT response (6, 7). One possible explanation for these findings is that the mechanical dyssynchrony caused by primary electric dyssynchrony is the modifiable substrate for CRT (8). The problem with conventional time-delay indices of mechanical dyssynchrony is that they can also be caused by regional contractile disparities such myocardial ischemia, infarction, or scar, which are less likely to be amenable to CRT (8, 9). As an alternative, visual assessments of apical rocking, septal flash, and left bundle branch block (LBBB) contraction pattern are used to assess left ventricular (LV) mechanical dyssynchrony, potentially overcoming the limitations of previously suggested parameters (10, 11).

In a healthy heart, all LV segments contract synchronously and myocardial energy is used efficiently to eject blood into the aorta. However, when there is a delay in electrical conduction, segments that activate early and late contract at different times, leading to the wastage of myocardial energy in stretching opposing walls. Several studies have shown that non-invasive estimates of global constructive work (CW) or wasted work (WW) using pressure–strain loops predict reverse remodeling or mortality after CRT better than dyssynchrony indices do (12–15). The combined assessment of myocardial CW and WW involves evaluating the contractile reserve and wasted energy caused by LV dyssynchrony, providing a comprehensive approach to evaluate the mechanisms underlying the CRT response. However, the prognostic value of regional CW and WW in CRT candidates has rarely been defined (16). In addition, a recent study showed that the combination of work difference between the septum and lateral wall (LW) with septal viability can be used to predict CRT response (17). The study employed cardiac magnetic resonance imaging with late gadolinium enhancement to evaluate septal viability. The current study aims to assess the efficacy of combining regional CW and WW in predicting reverse remodeling and clinical outcomes of patients undergoing CRT.

## Methods

### Study population

This was a prospective single-center study. We assessed patients with HF and LBBB who were undergoing CRT. We excluded patients who had atrial fibrillation, severe heart valve disease, or poor apical acoustic window. All patients were receiving optimized medical therapy at the time of CRT. An ischemic etiology was defined as a history of myocardial infarction, coronary revascularization, or angiographic evidence of multi-vessel disease or single-vessel disease with >75% stenosis of the left main or proximal left anterior descending artery. The study was approved by the institutional review board and complied with the Declaration of Helsinki. All patients provided written informed consent to participate in the study.

### Conventional echocardiographic analysis

All patients underwent transthoracic echocardiography using a commercially available ultrasound probe and device (M5S probe, Vivid E9, GE Healthcare, Horten, Norway) before and six months after CRT. Two dimensional and pulsed wave Doppler data were stored and analyzed offline. LV volumes and function were obtained using the modified Simpson's rule.

### Speckle tracking analysis

The study used digital loops of two-dimensional LV images for offline speckle-tracking analysis with a commercially available software (EchoPAC, GE Vingmed Ultrasound, Horten, Norway). The gain settings and sector width were adjusted to optimize the image quality with frame rates of 50–90 Hz. Two-dimensional LV images were obtained at the apical four-chamber, two-chamber, and long-axis views for speckle-tracking strain analysis. To analyze LV longitudinal strain, the endocardial border was traced on an end-systolic frame, and the width of the region of interest was adjusted to include most of the LV myocardium. The software automatically tracked myocardial motion and generated six curves of segmental longitudinal strain for each apical view. Global longitudinal strain was computed as the average of peak systolic longitudinal strain of all LV segments.

### Myocardial work assessment

The study utilized a vendor-specific software (EchoPAC version 202, GE Vingmed Ultrasound) to assess global and regional myocardial work. The peak LV pressure was assumed to be equivalent to the brachial systolic blood pressure, measured before the echocardiographic study. The software produced a previously validated noninvasive LV pressure curve that was adjusted based on the timing of ejection and isovolumic phases (18). These phases were defined by the timing of aortic valve and mitral valve opening and closing using spectral Doppler tracings. LV strain measured by speckle-tracking analysis and LV pressure curve were synchronized by aligning cardiac cycle phases and peak LV pressure. We quantified myocardial work by computing the rate of regional shortening via strain curve differentiation and multiplying this value by estimates of instantaneous LV pressure. Myocardial CW measurements quantified the amount of work performed during systolic shortening and the negative work performed while lengthening during isovolumic relaxation. Myocardial WW measurements quantified the amount of negative work performed while lengthening in systole and work performed while shortening in isovolumic relaxation. We computed regional CW and WW values for six regional walls (the inferior, posterior, lateral, anterior, anteroseptal, and septal walls) as the averages of the values for the basal- and mid-LV segments. We calculated the global values of CW and WW as the mean values for all LV walls.

### Alternative approaches

Two experienced observers assessed the existence of septal flash, apical rocking, and LBBB contraction pattern before CRT. Septal flash was characterized as the thickening and thinning of the septum during the isovolumic contraction period, while apical rocking was described as the movement of the LV apical myocardium vertical to the LV long axis (10). The LBBB contraction pattern was recognized by analyzing longitudinal strain curves in the apical four-chamber view using three criteria (11). These criteria included: (1) early shortening of at least one basal- or mid-LV segment in the septum and early lengthening in at least one basal- or mid-LV segment in the LW; (2) early septal peak shortening occurring within the initial 70% of the ejection period; and (3) LW reaching peak shortening after aortic valve closure (11). The work difference between the LW and septum was calculated in the apical four-chamber view as the absolute difference between net myocardial work in the LW and septum (17).

### Endpoints

The study's primary objective was to assess LV reverse remodeling, which was defined as a reduction in LV end-systolic volume (ESV) of ≥15% after six months of CRT. The secondary endpoint was the composite of all-cause death or hospitalization due to HF during a two-year follow-up.

### Statistical analysis

Continuous data are presented as mean ± standard deviation and categorical data as number and percentage. Comparisons among continuous variables were examined using the Student's *t*-test. Comparisons among categorical data were performed using the chi-squared test. We evaluate the predictive performance of global and regional CW and WW for reverse remodeling by calculating receiver-operating characteristic cures and areas under the curve (AUCs). To identify CRT responders, we selected an optimal cut-off value that maximized the Youden index (sensitivity + specificity − 1). Pearson's correlation analysis was conducted to examine the association between values of CW and WW and the decrease in LV ESV following CRT. To assess the predictive value of variables for reverse remodeling, we employed logistic regression analysis. Variables that had a univariate *p* value of <0.05 were included in a multivariate model. We utilized a series of nested models by incorporating CW (global or lateral) and WW (global or septal) parameters. The incremental predictive ability of each model was assessed by comparing chi-square values at each stage. To determine the cumulative probabilities of all-cause death or HF hospitalization after CRT, we employed the Kaplan–Meier method, and between-group comparisons of cumulative event rates were calculated using the log-rank test. We evaluate the inter- and intra-observer agreement for CW and WW in 20 randomly chosen patients. A *p-*value of <0.05 was considered statistically significant. We conduced statistical analysis using a statistical package (SPSS ver. 22.0, IBM, Chicago, IL, USA).

## Results

**Figure 1:**
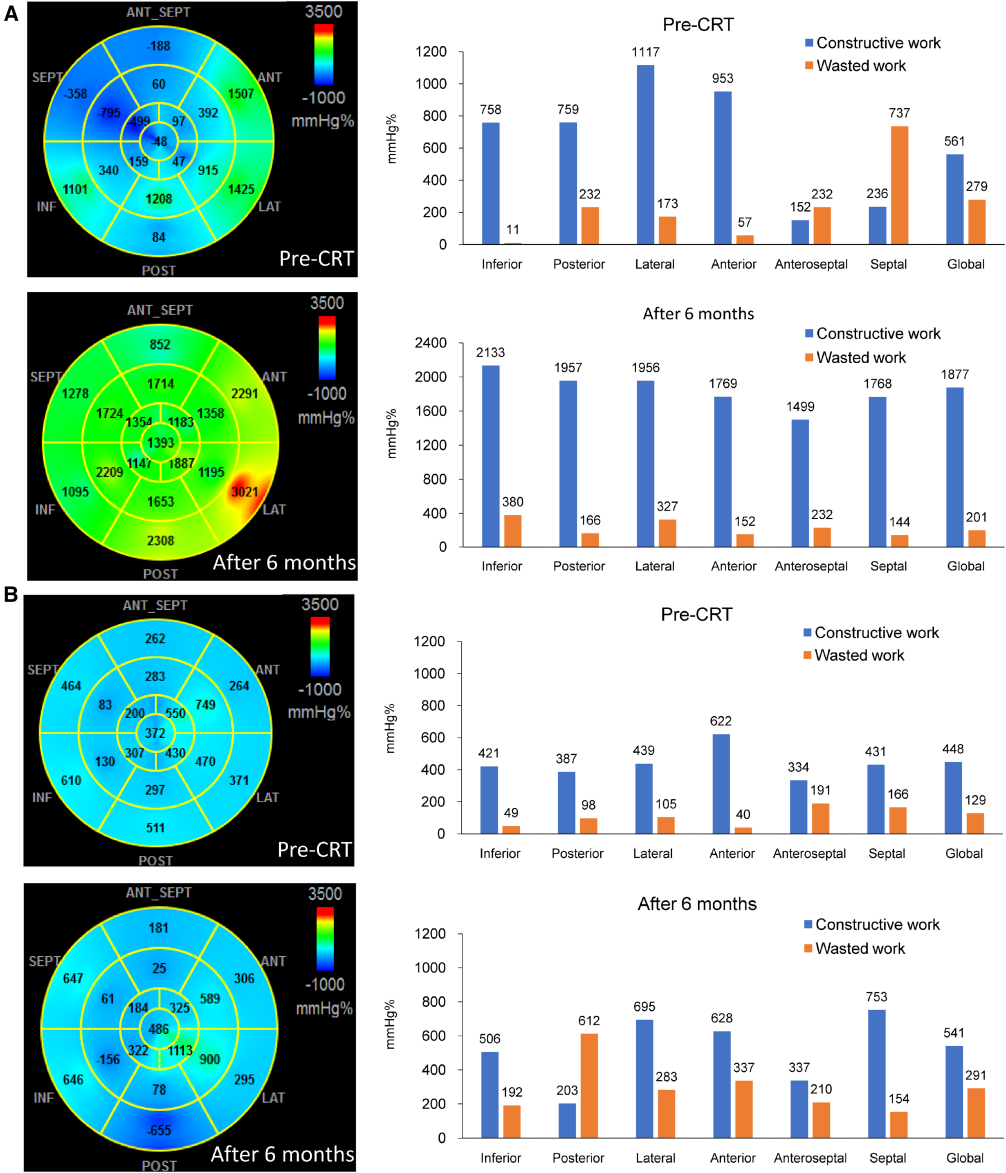
The bull-eye plots and bar charts showing the values of myocardial work, constructive work (CW) and wasted work (WW) before and 6 months after cardiac resynchronization therapy (CRT). In (Panel **A**), which represents a responder, high values of CW were observed in the lateral wall, while predominant WW was seen in the septum before CRT. Following CRT, there was a significant improvement in septal function, and the septal WW was converted to substantial CW. On the other hand, (Panel **B**), which represents a non-responder, showed lower values of lateral wall CW and septal WW compared to the responder. After CRT, there was only a moderate improvement in septal function, and noticeable WW was observed in the posterior wall.

Table 1 summarizes the baseline characteristics of the 134 patients included in the study, with an average age of 69.0 ± 11.9 years, 54.5% of whom were male, and 37.3% had ischemic etiology. Five patients died before the six-month follow-up and were classified as non-responders. Of the remaining 129 patients, 92 achieved the primary endpoint of a reduction in LV ESV of ≥15%, resulting in a response rate of 69%. Responders exhibited a higher prevalence of non-ischemic etiology, less dilated LV, and a more preserved LV ejection fraction and global longitudinal strain than non-responders. Prior to CRT, there were significant differences in regional CW between responders and non-responders in the posterior, lateral, anterior, and anteroseptal walls. There were also significant differences in regional WW in the anteroseptal and septal walls. Figure 1 displays the segmental values of myocardial work, CW, and WW in a responder (Panel A) and non-responder (Panel B) before CRT and after six months. Prior to CRT, the responder had marked differences in myocardial work, CW, and WW between regional walls, with large septal WW, which was converted to large CW with CRT. On the other hand, the non-responder shows smaller variations in myocardial work, CW, and WW before CRT. After CRT, there was only a modest improvement of septal function with noticeable WW in the posterior wall (LV pacing site). Supplementary Table S1 shows the effects of CRT on LV function and myocardial work. At follow-up, responders showed a significant improvement in LV ejection fraction and global longitudinal strain, whereas non-responders did not experience any changes in these parameters. Responders also exhibited significant improvements in septal WW, global CW, global WW, and work difference after six months, whereas non-responders did not show any significant changes in LW CW, septal WW, and global WW at follow-up.

**Table 1:** Baseline characteristics of the entire population and based on CRT response.

	All patients (*n *= 134)	Responders (*n *= 92)	Non-responders (*n* = 42)	*p*-value
Age, years	69.0 ± 11.9	69.9 ± 11.4	67.1 ± 12.9	0.214
Male	73 (54.5)	45 (48.9)	28 (66.7)	0.056
Ischemic etiology	50 (37.3)	23 (25)	27 (64.3)	<0.001
Medications				
ACE-inhibitor/ARB	105 (78.4)	73 (79.3)	32 (76.2)	0.681
ARNI	12 (9)	10 (10.9)	2 (4.8)	0.251
Beta-blocker	119 (88.8)	84 (91.3)	35 (83.3)	0.175
Aldosterone antagonist	73 (54.5)	53 (57.6)	20 (47.6)	0.281
QRS duration, ms	161.9 ± 19.3	162.2 ± 19.4	161.3 ± 19.3	0.812
eGFR, ml/min/1.73 m^2^	63.3 ± 31.1	65.1 ± 31.3	59.2 ± 30.8	0.309
QRS duration ≥150 ms	94 (70.1)	65 (70.7)	29 (69)	0.851
Systolic blood pressure, mmHg	120.1 ± 19.4	122.3 ± 19.4	115.4 ± 17.8	0.054
Diastolic blood pressure, mmHg	70.6 ± 12.0	71.2 ± 12.4	69.1 ± 10.8	0.343
NYHA class	2.8 ± 0.5	2.7 ± 0.5	2.9 ± 0.6	0.051
Mitral regurgitation	1.0 ± 0.7	1.0 ± 0.7	1.0 ± 0.7	0.787
LV EDV, ml	171.1 ± 67.6	160.0 ± 60.1	195.6 ± 76.8	0.004
LV ESV, ml	132.8 ± 62.6	122.4 ± 55.1	155.5 ± 72.0	0.004
LV ejection fraction, %	24.6 ± 7.5	25.6 ± 6.8	22.3 ± 8.5	0.020
GLS, %	−6.5 ± 2.9	−7.0 ± 2.9	−5.3 ± 2.7	0.001
Septal flash	90 (67.2)	75 (81.5)	15 (35.7)	<0.001
Apical rocking	97 (72.4)	80 (87.0)	17 (40.5)	<0.001
LBBB contraction pattern	88 (65.7)	75 (81.5)	13 (31.0)	<0.001
Work difference, mmHg%	953 ± 530	1,115 ± 498	596 ± 415	<0.001
Global CW, mmHg%	767 ± 346	846 ± 346	592 ± 277	<0.001
Global WW, mmHg%	279 ± 148	301 ± 149	230 ± 135	<0.001
Regional CW, mmHg%				
Inferior wall	636 ± 384	634 ± 383	642 ± 392	0.911
Posterior wall	1,103 ± 548	1,263 ± 525	752 ± 423	<0.001
Lateral wall	1,026 ± 493	1,175 ± 467	701 ± 381	<0.001
Anterior wall	853 ± 415	943 ± 406	655 ± 365	<0.001
Anteroseptum	486 ± 337	527 ± 364	396 ± 250	0.017
Septum	407 ± 302	402 ± 324	418 ± 249	0.779
Regional WW, mmHg%				
Inferior wall	261 ± 204	283 ± 203	215 ± 201	0.076
Posterior wall	313 ± 204	332 ± 202	272 ± 207	0.115
Lateral wall	255 ± 164	246 ± 151	273 ± 190	0.379
Anterior wall	182 ± 136	175 ± 121	197 ± 164	0.383
Anteroseptum	298 ± 286	341 ± 306	204 ± 212	0.003
Septum	407 ± 280	474 ± 278	261 ± 226	<0.001

ACE, angiotensin converting enzyme; ARB, angiotensin receptor blocker; ARNI, angiotensin receptor neprilysin inhibitor; CW, constructive work; EDV, end-diastolic volumn; eGFR, estimated glomerular filtration rate; ESV, end-systolic volumn; GLS, global longitudinal strain; LBBB, left bundle branch block; LV, left ventricular; NYHA, New York Heart Association; WW, wasted work.

Data are shown as *n* (%) or mean ± standard deviation.

### Predictive characteristics for reverse remodeling after CRT

**Figure 2:**
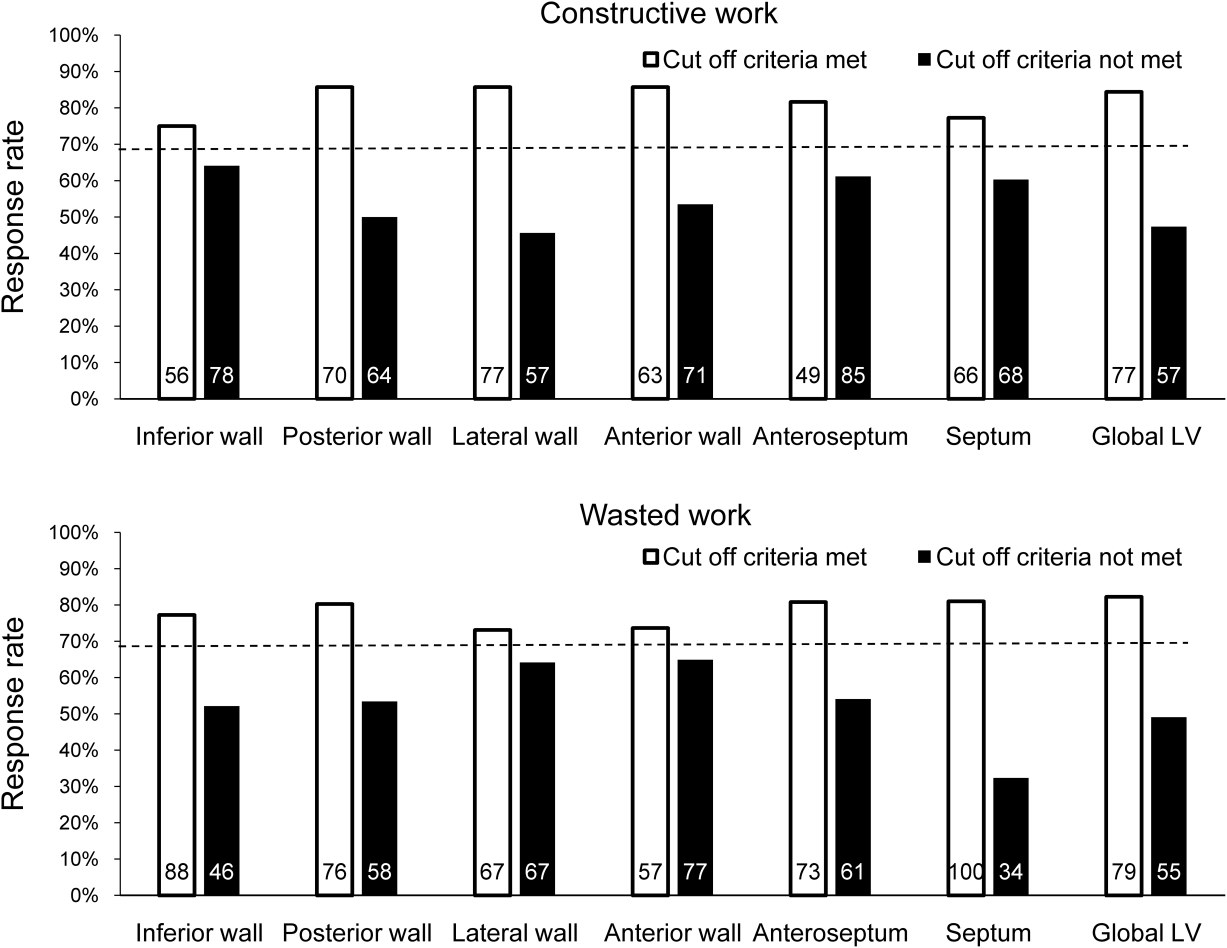
Response rate at 6 months after CRT by determining whether the cut-off value for each CW and WW parameter was met in all patients. The dashed line indicates the response rate observed when ignoring the parameter. The number of patients who met or did not meet the cut-off criterion for each parameter is shown inside each bar.

Based on the binary definition of reverse remodeling, posterior wall CW, LW CW, anterior wall CW, anteroseptal WW, and septal WW had an AUC greater than that under the line of no information (Table 2). Of the regional CW values, the LW CW varied the most between responders and non-responders [AUC: 0.783, 95% confidence interval (CI) 0.700–0.866, cut-off value 878 mmHg%, sensitivity 72%, specificity 74%]. Of the regional WW values, septal WW varied the most between responders and non-responders (AUC: 0.737, 95% CI: 0.644–0.831, cut-off value: 181 mmHg%, sensitivity 88%, specificity 55%). Combining LW CW and septal WW increased the AUC to 0.832 (95% CI: 0.755–0.908). Figure 2 displays the response rates of patients whose regional CW and WW values met (true-positive rate) or did not meet (false-negative rate) the cut-off values. The results show that LW CW was superior to the other regional CW measures and global CW, with a true-positive rate of 86% and a false-negative rate of 46%. Septal WW was superior to the other regional WW measures and global WW, with a true-positive rate of 81% and a false-negative rate of 32%. The AUCs for global CW and WW were 0.732 (95% CI: 0.639–0.825) and 0.692 (95% CI: 0.589–0.796), respectively. Combining the global CW and WW increased the AUC to 0.759 (95% CI: 0.669–0.850).

**Table 2:** Predictive characteristics of regional constructive work and wasted work prior to cardiac resynchronization therapy.

	Versus ΔESV		Dichotomous reverse remodeling response			
	CC	*p*-value	AUC (95% CI)	Cut-off, mmHg%	Sensitivity, %	Specificity, %
Constructive work						
Inferior wall	0.01	0.950	0.507 (0.400–0.614)	488	67	46
Posterior wall	0.38	<0.001	0.774 (0.692–0.857)	1,052	65	76
Lateral wall	0.35	<0.001	0.783 (0.700–0.866)	878	72	74
Anterior wall	0.23	0.009	0.720 (0.624–0.816)	822	59	79
Anteroseptum	0.05	0.598	0.602 (0.502–0.702)	559	44	79
Septum	−0.14	0.118	0.557 (0.451–0.663)	333	64	55
Global LV	0.24	0.007	0.732 (0.639–0.825)	635	71	71
Wasted work						
Inferior wall	0.07	0.397	0.617 (0.510–0.724)	136	74	52
Posterior wall	0.11	0.206	0.619 (0.510–0.729)	236	66	64
Lateral wall	−0.11	0.196	0.529 (0.422–0.636)	232	57	53
Anterior wall	−0.06	0.532	0.514 (0.408–0.621)	132	64	46
Anteroseptum	0.24	0.005	0.650 (0.552–0.748)	172	64	67
Septum	0.33	<0.001	0.737 (0.644–0.831)	181	88	55
Global LV	0.18	0.039	0.692 (0.589–0.796)	222	71	67

AUC, area under the curve; CC, correlation coefficient; ESV, end-systolic volume; LV, left ventricle.

### Variables associated with reverse remodeling

**Figure 3:**
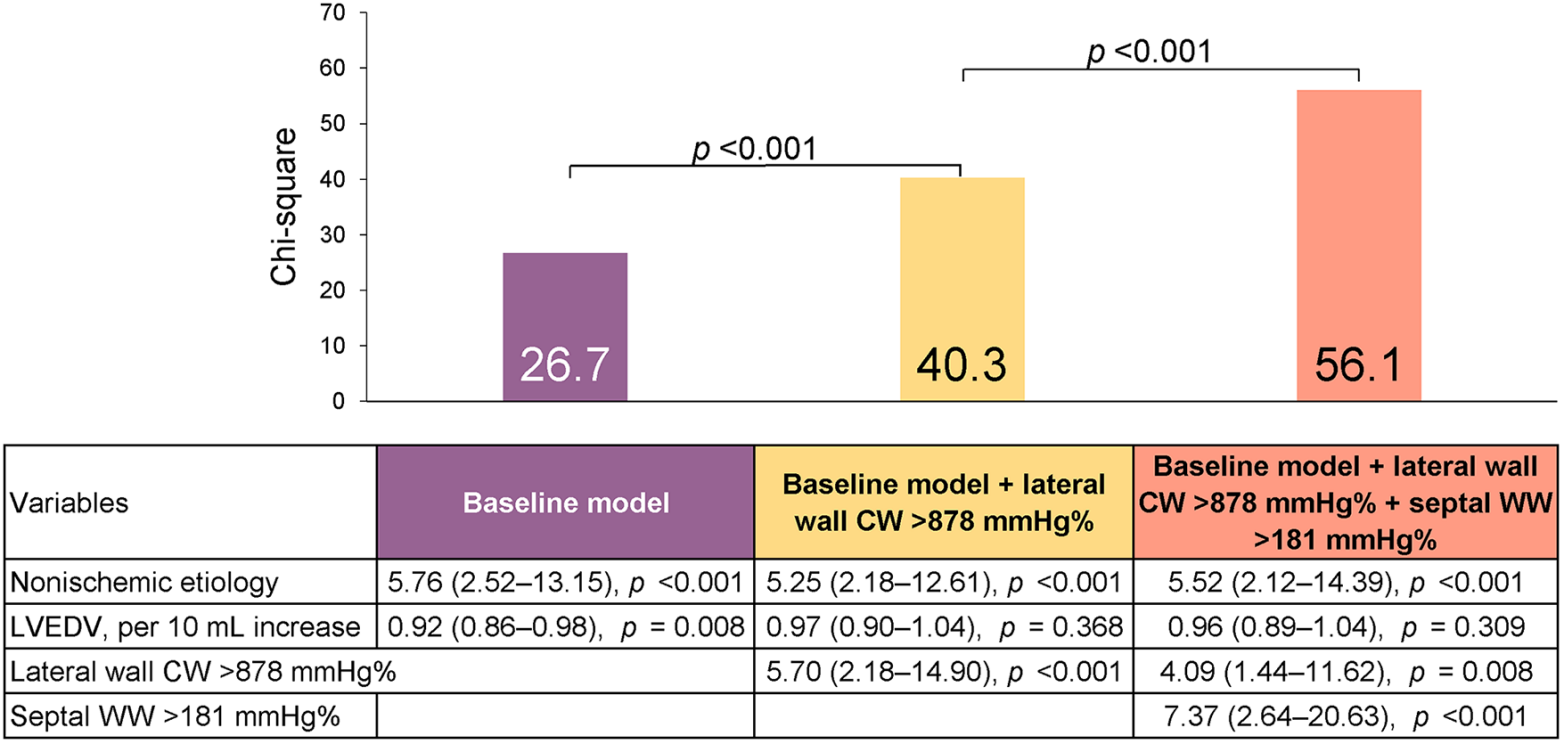
Predicting reverse remodeling after CRT. Model *χ*^2^ values are presented for a series of nested models. The baseline model included non-ischemic etiology and left ventricular end-diastolic volume (LVEDV).

Multivariate analysis, using the significant variables from the univariate analysis (Supplementary Table S2) revealed that non-ischemic etiology and LV end-diastolic volume were independently associated with reverse remodeling, and they were thus included in the baseline model (*χ*^2^ = 26.7, Table 3). We then added the CW (lateral and global) and/or WW (septal and global) parameters to the model. The LW CW [odds ratio (OR) 1.26, 95% CI: 1.10–1.44 per 100-mmHg% increase] and septal WW (OR 1.33, 95% CI: 1.07–1.66 per 100-mmHg% increase) were both independently associated with reverse remodeling. Model power improved when LW CW (*χ*^2^ difference: 24.4, *p *< 0.001) and septal WW (*χ*^2^ difference 17.2, *p *< 0.001) were added to the model. In contrast, global CW and WW were not independently associated with reverse remodeling when LW CW or septal WW was included in the model. The addition of LW CW >878 mmHg% (OR 4.09; 95% CI: 1.44–11.62) and septal WW >181 mmHg% (OR 7.37; 95% CI: 2.64–20.63) to a baseline model including non-ischemic etiology and LV end-diastolic volume significantly increased model power (Figure 3). There were 66 patients (49%) with both LW CW >878 mmHg% and septal WW >181 mmHg%. Of this group, 29% (*n *= 19) showed ischemic cardiomyopathy, which was a significantly smaller proportion than was observed in the other group (*p *= 0.044). This presence of both LW CW >878 mmHg% and septal WW >181 mmHg% was associated with a high response rate (91%). There were 23 patients (17%) with both LW CW ≤878 mmHg% and septal WW ≤181 mmHg%. Their response rate was only 21%. The response rate in the 45 patients (34%) who had either LW CW >878 mmHg% or septal WW >181 mmHg% was 60%.

**Table 3:** Variables associated with CRT response in the baseline model and after addition of constructive work and wasted work parameters.

	Baseline model		Baseline model + global CW + global WW		Baseline model + global CW + LW CW		Baseline model + global WW + septal WW		Baseline model + LW CW + septal WW	
	OR (95% CI)	*p*-value	OR (95% CI)	*p*-value	OR (95% CI)	*p*-value	OR (95% CI)	*p*-value	OR (95% CI)	*p*-value
Non-ischemic etiology	5.76 (2.52–13.15)	<0.001	6.62 (2.67–16.41)	<0.001	6.65 (2.61–16.94)	<0.001	4.99 (2.06–12.12)	<0.001	5.64 (2.16–14.73)	<0.001
LVEDV, per 10 ml	0.92 (0.86–0.98)	0.008	0.97 (0.91–1.04)­	0.433	0.98 (0.91–1.05)	0.486	0.91 (0.85–0.97)	0.005	0.96 (0.90–1.03)	0.293
Global CW per 100-mmHg%			1.32 (1.09–1.56)	0.005	0.89 (0.67–1.18)	0.399				
Global WW per 100-mmHg%			1.46 (1.00–2.13)	0.049			0.84 (0.50–1.42)	0.518		
LW CW, per 100-mmHg%					1.41 (1.15–1.72)	0.001			1.26 (1.10–1.44)	0.001
Septal WW, per 100-mmHg%							1.54 (1.17–2.04)	0.002	1.33 (1.07–1.55)	0.011

CI, confidence interval; CW, constructive work; LVEDV, left ventricular end-diastolic volume; LW, lateral wall; OR, odds ratio; WW, wasted work.

### Event-free survival

**Figure 4:**
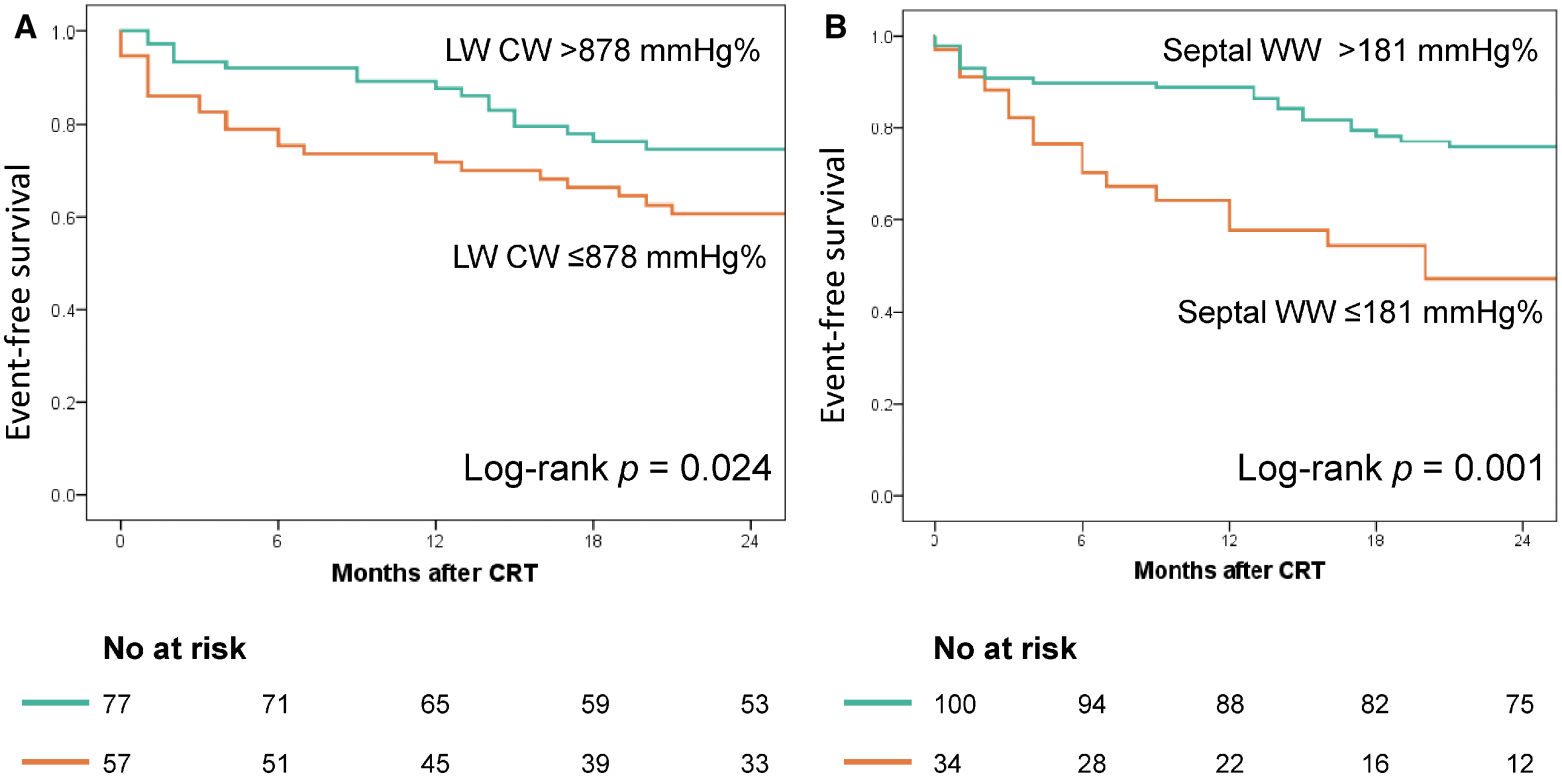
Association of lateral wall (LW) CW and septal WW with event-free survival. The Kaplan–Meier curves were stratified based on the cut-off values for LW CW (878 mmHg%, Panel **A**) and septal WW (181 mmHg%, Panel **B**).

**Figure 5:**
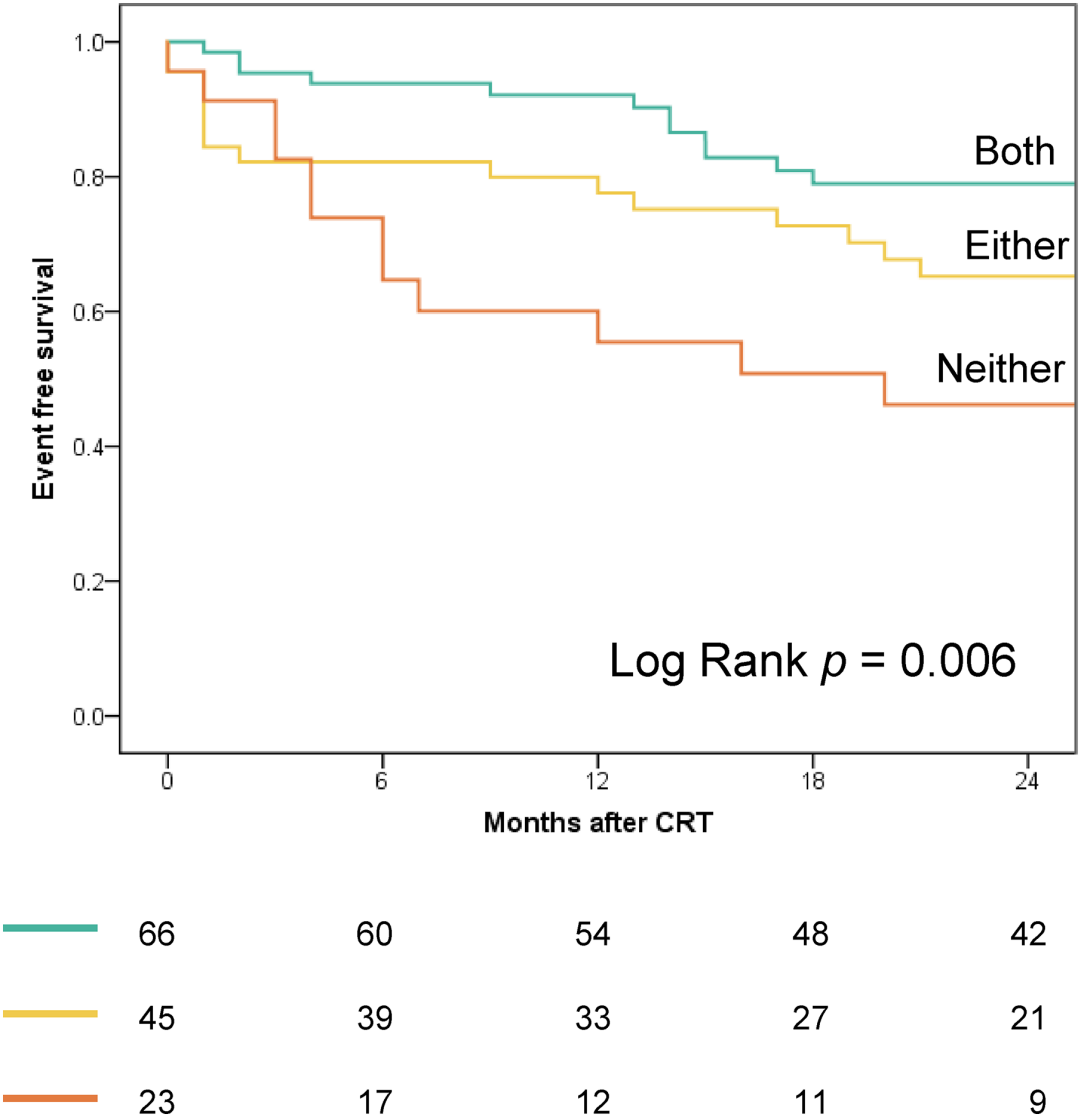
Kaplan–Meier curves, stratified by combined LW CW and septal WW parameters, depict outcomes for combined all-cause death and heart failure hospitalization. “Both” group: LW CW >878 mmHg% and septal WW >181 mmHg; “Either” group: LW CW >878 mmHg% or septal WW >181 mmHg (but not both); “Neither” group: Neither CW >878 mmHg% nor septal WW >181 mmHg.

Figure 4 displays the Kaplan–Meier curves dichotomized according to LW CW ≤878 mmHg% (log-rank *p* = 0.024) and septal WW ≤181 mmHg% (log-rank *p* = 0.001). Both CW ≤878 mmHg% (HR 2.01; 95% CI: 1.07–3.79, *p* = 0.031) and septal WW ≤181 mmHg% (HR 2.60; 95% CI: 1.38–4.90; *p* = 0.003) were significant predictors of combined all-cause death and hospitalization due to HF at two-year follow-up. Figure 5 displays the Kaplan–Meier curves stratified by the combined LW CW and septal WW parameters. Patients categorized in the “both” group, characterized by both LW CW >878 mmHg% and septal WW >181 mmHg%, demonstrated the most favorable outcomes in terms of combined all-cause death and HF hospitalization. Conversely, individuals in the “neither” group, characterized by neither LW CW >878 mmHg% nor septal WW >181 mmHg %, exhibited the worst outcomes. Patients in the “either” group, with only one parameter meeting the criteria, were positioned between the “both” and “neither” groups in terms of their outcomes.

### Alternative approaches

Septal flash, apical rocking, and LBBB strain pattern predicted reverse remodeling with AUC values of 0.729 (95% CI: 0.632–0.826), 0.732 (95% CI: 0.633–0.832), and 0.753 (95% CI: 0.659–0.847), respectively (Table 4). There were no significant differences when comparing the AUC for work difference (0.780; 95% CI: 0.698–0.863) with septal flash (*p* = 0.291) or apical rocking (*p* = 0.386). In contrast, the combination of LW CW and septal WW (AUC: 0.832; 95% CI: 0.755–0.908) was superior to septal flash (*p* = 0.029) and apical rocking (*p* = 0.035) in predicting reverse remodeling. Furthermore, in multivariate logistic regression analysis, combining LW CW and septal WW (odds ratio 1.23; 95% CI: 1.02–1.49) but not work difference (odds ratio 0.90; 95% CI: 0.72–1.13) was an independent factor associated with reverse remodeling (Table 5).

**Table 4:** Comparison of the area under the curves for predicting reverse remodeling after CRT.

		Compared with work difference		Compared with lateral CW + 1.5 × septal WW	
	AUC (95% CI)	Difference in AUC (95% CI)	*p*-value	Difference in AUC (95% CI)	*p*-value
Septal flash	0.729 (0.632–0.826)	0.051 (−0.044 to 0.146)	0.291	0.102 (0.011 to 0.194)	0.029
Apical rocking	0.732 (0.633–0.832)	0.048 (−0.060 to 0.156)	0.386	0.099 (0.007 to 0.191)	0.035
LBBB strain pattern	0.753 (0.659–0.847)	0.027 (−0.060 to 0.115)	0.541	0.079 (−0.008 to 0.165)	0.075
Work difference	0.780 (0.698–0.863)	—		0.051 (−0.006 to 0.108)	0.077
LW CW + 1.5 × septal WW	0.832 (0.755–0.908)	—		—	

LW, lateral wall; CW, constructive work; WW, wasted work.

**Table 5:** Multivariate logistic regressiona analysis with LV reverse remodeling as dependent variable.

Regression variable	OR	95% CI	*p*-value
LV end-diastolic volume per 10-ml increase	0.93	0.86–1.01	0.067
Non-ischemic etiology	3.88	1.30–11.59	0.015
Septal flash	1.76	0.56–5.55	0.337
Apical rocking	2.42	0.70–8.35	0.161
LBBB strain pattern	3.66	1.05–12.79	0.042
Work difference, per 100-mmHg% increase	0.90	0.72–1.13	0.364
LW CW + 1.5 × septal WW, per 100-mmHg% increase	1.23	1.02–1.49	0.033

CI, confidence interval; CW, constructive work; LW, lateral wall; OR, odds ratio; WW, wasted work.

*N* = 134, Cox and Snell *R*^2^ = 0.415.

### Signs of mechanical dyssynchrony and LW CW & septal WW

Patients with septal flash exhibited significantly elevated LW CW values (1,113 ± 459 mmHg% vs. 848 ± 517 mmHg%, *p* < 0.001) and septal WW values (486 ± 271 mmHg% vs. 245 ± 225 mmHg%, *p* = 0.003) compared to those without this characteristic. Similarly, patients with apical rocking displayed notably higher LW CW values (1,095 ± 459 mmHg% vs. 846 ± 537 mmHg%, *p* < 0.001) and septal WW values (488 ± 275 mmHg% vs. 193 ± 149 mmHg%, *p* = 0.008) than those without.

### Non-ischemic and ischemic patient subgroups

There were no significant differences in LW CW (1,057 ± 471 mmHg% vs. 975 ± 527 mmHg%; *p *= 0.353) and septal WW (438 ± 278 mmHg% vs. 355 ± 280 mmHg%; *p *= 0.098) between non-ischemic and ischemic patients. In patients with non-ischemic etiology, LW CW and septal WW were correlated with the reductions in LV ESV and had high AUC values (LW CW: 0.827, 95% CI: 0.693–0.961; septal WW: 0.761, 95% CI: 0.627–0.896; Supplementary Table S3). Including both LW CW and septal WW in the model increased the AUC to 0.875 (95% CI: 0.753–0.998). In patients with ischemic etiology, LW CW rather than septal WW was correlated with reductions in LV ESV. LW CW (AUC: 0.749; 95% CI: 0.617–0.892) and septal WW (AUC: 0.704; 95% CI: 0.559–0.848) varied between responders and non-responders. Including both LW CW and septal WW in the model increased the AUC to 0.771 (95% CI: 0.642–0.901).

### Inter- and intra-observer variability and reproducibility

Calculations of LW CW and septal WW in 20 patients by two independent observers differed on average by 109 mmHg% and 74 mmHg%, respectively. Repeat calculations these measures by the same observer differed on average by 95 mmHg% and 57 mmHg%, respectively. The intraclass correlation coefficient between the two observers was 0.96 (95% CI: 0.89–0.98) and 0.97 (95% CI: 0.92–0.99) for LW CW and septal WW, respectively. The intra-observer intraclass correlation coefficient was 0.98 (95% CI: 0.95–0.99) and 0.97 (95% CI: 0.94–0.99) for LW CW and septal WW, indicating good reproducibility.

## Discussion

This study extends prior researches on myocardial work and presents the novel finding that the assessment of regional CW and WW via non-invasive pressure–strain loops can offer valuable prognostic insights for individuals who were being considered for CRT. Prior to CRT, LW CW and septal WW were significantly correlated with the reductions in LV ESV after CRT and independently predicted reverse remodeling and clinical outcomes after CRT. Global CW and WW were similarly correlated with the extent of reverse remodeling; however, they did not independently predict reverse remodeling when LW CW and septal WW were taken into account. The latter two measures were useful for predicting CRT response among both ischemic and non-ischemic patients.

The rationale for using LW CW and septal WW to predict CRT outcomes is that electrical conduction delay in the failing heart provokes discoordinate contraction between the early-activated septum and the late-activated LW. In patients with HF and LBBB, the ventricular septum contracts early during the isovolumic contraction phase, and during ejection, the out-of-phase septal relaxation counteracts LV free wall contraction. Regional CW quantifies the work performed during systolic shortening and negative work while lengthening during isovolumic relaxation, and reflects the contractile reserve. Regional WW computes the amount of negative work performed while lengthening during systole and work performed while shortening during isovolumic relaxation, and reflects energy waste caused by mechanical dyssynchrony. CRT can recruit myocardial work that is internally wasted by discoordinate contraction, and assessing the LW CW and septal WW facilitates identification of the contractile reserve and recruitable substrate that are amenable to CRT.

Previous studies have shown the prognostic value of global CW and WW in CRT candidates (12–15). In a study of 97 patients undergoing CRT, global CW was associated with CRT response and was significantly correlated with the reductions in LV ESV after CRT (12). Despite higher values of LW CW and septal WW in responders, neither measure was independently associated with CRT response after adjusting for global CW and septal flash (12). Two studies have shown the ability of global WW to predict response to CRT (14, 15). One study found that combining global CW greater than 1,057 mmHg% and global WW greater than 364 mmHg% had a high specificity but low sensitivity for predicting CRT response (14). Another study involving 249 patients with HF found that a pre-CRT GWW of less than 200 mmHg% was associated with a high risk of all-cause mortality and CRT non-response (15). In our study, higher values of global CW and WW before CRT were associated with CRT response. Of the regional CW and WW values, LW CW and septal WW best distinguished CRT responders from non-responders. LW CW and septal WW performed better than global CW and global WW, respectively, with respect to reverse remodeling. This finding differs from the aforementioned study by Galli et al. (12). One possible reason for this discrepancy is that global measures of CW and WW, derived from the average of all segments, may lose significant information that is embedded in the nonhomogeneous distribution of regional CW and WW in CRT candidates. Our results agree with the results of two other studies that separately showed the prognostic value of LW CW or septal WW in patients undergoing CRT (16, 19). In a brief report on 168 CRT candidates, LW CW rather than septal WW was independently associated with CRT response, and a LW CW >881 mmHg% was associated with a 2.2-fold increase in CRT response odds (16). In a small study of 21 patients receiving CRT, septal WW rather than global WW was the only myocardial work factor that predicted LV ESV reductions after CRT (19). However, the definition of septal WW, negative work in percentage of positive work, differs from ours (19).

In the present study, we found that septal WW was less related to reverse remodeling after CRT in patients with ischemic cardiomyopathy. Distinguishing between systolic lengthening of the septum due to transmural scar and septal systolic stretching resulting from LBBB, presents a challenge. Consequently, the similarity in septal WW between patients with myocardial scar and patients with electrical conduction delay may account for the weaker association of septal WW with reverse remodeling after CRT in patients with ischemic cardiomyopathy. The considerable variability in the extent of septal WW among patients with LBBB likely reflects, at least in part, this mixed etiology of systolic lengthening.

The use in clinical practice of myocardial work assessment derived from non-invasive pressure–strain loops for prognostic and clinical decision-making purposes is increasing (17, 20–26). The reliability of non-invasive measures of myocardial work in comparison to invasive measures has been validated in experimental evaluations and computer simulations (18, 27). In the present study, the combined approach of LW CW and septal WW offers a clinically feasible and relatively simple method for identifying CRT responders. Both parameters were measured from the basal- and mid-segments of the LW and septum in the apical four-chamber view, which can be obtained for all patients. Evaluating LW CW and septal WW incorporates the assessment of contractile reserve and energy waste, which are key factors determining the response to CRT.

### Limitations

This study has some limitations. Firstly, it is a single-center study, which may limit the generalization of its findings to clinical practice. Secondly, the lack of a validation cohort to examine the results further limits its generalizability. Thirdly, to assess myocardial work, a vendor-specific module (EchoPAC, GE) that combines LV strain data with a non-invasive LV pressure curve is required. Lastly, the study did not evaluate septal viability, and it is unclear whether it provides additional valve over septal WW and LW CW. Further study may be needed to address this issue.

## Conclusion

This study revealed that LW CW and septal WW before CRT, assessed based on pressure–strain loops, predicted reverse remodeling and clinical outcomes after CRT. These two measurements reliably identified potential CRT responders in both ischemic and non-ischemic patients, and may better identifying CRT responders than the work difference between septum and LW.

## Data Availability

The raw data supporting the conclusions of this article will be made available by the authors, without undue reservation.
