# Supplementary material for: Clinical significance of regional constructive and wasted work in patients receiving cardiac resynchronization therapy
Source: Front Cardiovasc Med. 2024 Mar 6;11:1301140. doi: 10.3389/fcvm.2024.1301140 (PMC10950977; doi:10.3389/fcvm.2024.1301140)
Supplement: Supplementary file 1 [file Table1.docx]

**SUPPLEMENTARY TABLE S1** Changes in left ventricular function and myocardial work after 6 months of cardiac resynchronization therapy

|  |  | **Responders (*n* = 92)** | | |  | **Non-responders* (*n* = 37)** | | |
| --- | --- | --- | --- | --- | --- | --- | --- | --- |
|  |  | **Pre-CRT** | **6 months** | ***p*-value** |  | **Pre-CRT** | **6 months** | ***p*-value** |
| LV end-diastolic volume, ml |  | 160.0 ± 60.1 | 112.4 ± 48.0 | <0.001 |  | 194.5 ± 63.7 | 208.1 ± 70.1 | 0.009 |
| LV end-systolic volume, ml |  | 122.4 ± 55.2 | 65.71 ± 42.1 | <0.001 |  | 154.0 ± 59.8 | 163.7 ± 62.4 | 0.020 |
| LV ejection fraction, % |  | 25.6 ± 6.9 | 44.1 ± 11.9 | <0.001 |  | 22.3 ± 8.5 | 22.5 ± 8.6 | 0.845 |
| Global longitudinal strain, % |  | -7.0 ± 2.9 | -10.3 ± 3.4 | <0.001 |  | -5.2 ± 2.8 | -5.7 ± 3.1 | 0.313 |
| Lateral wall CW, mmHg% |  | 1179 ± 476 | 1127 ± 281 | 0.420 |  | 676 ± 371 | 567 ± 309 | 0.105 |
| Septal WW, mmHg% |  | 300 ± 149 | 180 ± 97 | <0.001 |  | 263 ± 222 | 124 ± 115 | <0.001 |
| Global CW, mmHg% |  | 852 ± 352 | 1278 ± 448 | <0.001 |  | 588 ± 284 | 715 ± 399 | 0.032 |
| Global WW, mmHg% |  | 300 ± 149 | 180 ± 97 | <0.001 |  | 229 ± 140 | 194 ± 91 | 0.070 |
| Work difference, mmHg% |  | 1115 ± 403 | 436 ± 403 | <0.001 |  | 584 ± 404 | 307 ± 279 | 0.003 |

Data are presented as mean ± standard deviation.

*Five patients who died before six-month follow-up were not included in the analysis.

CRT, cardiac resynchronization therapy; CW, constructive work; LV, left ventricular; WW, wasted work.

**SUPPLEMENTARY TABLE S2** Univariate logistic regression analysis showing the variables associated with left ventricular reverse remodeling after cardiac resynchronization therapy

| Univariate analysis | **OR (95% CI)** | ***p*-value** |
| --- | --- | --- |
| Age, per 10 years | 1.22 (0.89–1.66) | 0.214 |
| Female | 2.09 (0.98–4.47) | 0.058 |
| LV end-diastolic volume, per 10 mL* | 0.93 (0.87–0.98) | 0.007 |
| LV end-systolic volume, per 10 mL | 0.92 (0.87–0.98) | 0.007 |
| LV ejection fraction, per 1% | 1.06 (1.01–1.12) | 0.022 |
| Global longitudinal strain, per 1% | 0.79 (0.68–0.92) | 0.002 |
| Nonischemic etiology* | 5.40 (2.46–11.88) | <0.001 |
| Estimated glomerular filtration rate, per  10 mL/min/1.73m^2^ | 1.10 (0.97–1.25) | 0.146 |
| QRS duration, per 10 ms | 1.02 (0.85–1.24) | 0.810 |
| QRS duration >150 ms | 1.08 (0.49–2.39) | 0.851 |
| Posterior wall CW, per 100-mmHg% | 1.26 (1.14–1.39) | <0.001 |
| Lateral wall CW, per 100-mmHg% | 1.32 (1.18–1.48) | <0.001 |
| Anterior wall CW, per 100-mmHg% | 1.23 (1.10–1.39) | <0.001 |
| Global CW, per 100-mmHg% | 1.34 (1.15–1.56) | <0.001 |
| Anteroseptal WW, per 100-mmHg% | 1.26 (1.05–1.51) | 0.014 |
| Septal WW, per 100-mmHg% | 1.45 (1.20–1.51) | <0.001 |
| Global WW, per 100-mmHg% | 1.57 (1.12–2.21) | 0.009 |
| Lateral wall CW >878 mmHg% | 7.15 (3.14–16.31) | <0.001 |
| Septal WW >181 mmHg% | 8.91 (3.72–21.39) | <0.001 |

CI, confidence interval; CW, constructive work; OR, odds ratio; WW, wasted work.

*Variables in the baseline model.

**SUPPLEMENTARY TABLE S3** Predictive characteristics of baseline CW and WW in non-ischemic and ischemic patients

|  | **Versus ΔESV** | |  | **Dichotomous reverse remodeling response** | | | |
| --- | --- | --- | --- | --- | --- | --- | --- |
|  | **CC** | **p-value** |  | **AUC (95% CI)** | **Cutt-off mmHg%** | **Sensitivity (%)** | **Specificity (%)** |
| **Non-ischemic cardiomyopathy (n = 84)** | | | | |  |  |  |
| Constructive work, mmHg% | | |  |  |  |  |  |
| Inferior wall | -0.01 | 0.960 |  | 0.545 (0.366–0.724) | 348 | 77 | 40 |
| Posterior wall | 0.36 | 0.001 |  | 0.858 (0.761–0.955) | 781 | 83 | 73 |
| Lateral wall | 0.30 | 0.006 |  | 0.827 (0.693–0.961) | 673 | 88 | 73 |
| Anterior wall | 0.16 | 0.145 |  | 0.767 (0.620–0.914) | 651 | 71 | 73 |
| Anteroseptum | -0.01 | 0.949 |  | 0.605 (0.468–0.742) | 329 | 67 | 60 |
| Septum | -0.14 | 0.213 |  | 0.528 (0.360–0.696) | 310 | 69 | 59 |
| Global LV | 0.12 | 0.299 |  | 0.752 (0.587–0.918) | 564 | 75 | 80 |
| Wasted work, mmHg% | | |  |  |  |  |  |
| Inferior wall | 0.11 | 0.329 |  | 0.608 (0.452–0.764) | 132 | 74 | 47 |
| Posterior wall | 0.03 | 0.767 |  | 0.534 (0.357–0.712) | 252 | 62 | 67 |
| Lateral wall | -0.03 | 0.758 |  | 0.525 (0.369–0.681) | 109 | 100 | 16 |
| Anterior wall | 0.01 | 0.995 |  | 0.523 (0.379–0.668) | 141 | 73 | 48 |
| Anteroseptum | 0.24 | 0.027 |  | 0.669 (0.523–0.814) | 172 | 64 | 73 |
| Septum | 0.24 | 0.026 |  | 0.761 (0.627–0.896) | 181 | 88 | 60 |
| Global LV | 0.18 | 0.039 |  | 0.731 (0.588–0.874) | 243 | 62 | 80 |
| **Ischemic cardiomyopathy (n = 50)** | | |  |  |  |  |  |
| Constructive work | | |  |  |  |  |  |
| Inferior wall | 0.17 | 0.248 |  | 0.528 (0.362–0.694) | 881 | 35 | 82 |
| Posterior wall | 0.46 | 0.001 |  | 0.754 (0.617–0.892) | 1055 | 74 | 70 |
| Lateral wall | 0.40 | 0.004 |  | 0.749 (0.611–0.887) | 1208 | 52 | 89 |
| Anterior wall | 0.32 | 0.027 |  | 0.717 (0.572–0.861) | 667 | 83 | 56 |
| Anteroseptum | 0.15 | 0.316 |  | 0.634 (0.480–0.789) | 537 | 57 | 74 |
| Septum | 0.03 | 0.825 |  | 0.525 (0.361–0.689) | 515 | 52 | 67 |
| Global LV | 0.40 | 0.005 |  | 0.753 (0.616–0.890) | 878 | 52 | 89 |
| Wasted work |  |  |  |  |  |  |  |
| Inferior wall | 0.07 | 0.625 |  | 0.625 (0.469–0.781) | 136 | 74 | 56 |
| Posterior wall | 0.07 | 0.645 |  | 0.651 (0.495–0.806) | 218 | 65 | 70 |
| Lateral wall | -0.15 | 0.306 |  | 0.502 (0.339–0.666) | 408 | 26 | 91 |
| Anterior wall | -0.08 | 0.586 |  | 0.519 (0.356–0.683) | 266 | 33 | 83 |
| Anteroseptum | 0.18 | 0.222 |  | 0.652 (0.499–0.806) | 168 | 70 | 63 |
| Septum | 0.17 | 0.237 |  | 0.704 (0.559–0.848) | 155 | 96 | 44 |
| Global LV | 0.18 | 0.039 |  | 0.671 (0.519–0.824) | 197 | 83 | 59 |

AUC, area under the curve; CC, correlation coefficient; CI, confidence interval; ΔESV: change in end-systolic volume; LV, left ventricle.
